# Benefits of Nutrition Education in Local Community Supported Agriculture Sites: A Case Study

**DOI:** 10.3390/ijerph22071033

**Published:** 2025-06-28

**Authors:** Bree Bode, Sarah Mott, Jacob M. Cutler, Nicole Jess, Sarah Panken, Marci Scott

**Affiliations:** Michigan Fitness Foundation, 2843 Eyde Pkwy, East Lansing, MI 48823, USA; smott@michiganfitness.org (S.M.); jcutler@michiganfitness.org (J.M.C.); njess@michiganfitness.org (N.J.); slpanken@michiganfitness.org (S.P.); mscott@michiganfitness.org (M.S.)

**Keywords:** nutrition education, nutrition incentives, supplemental nutrition assistance program, supplemental nutrition assistance program education, community supported agriculture, food security, nutrition security

## Abstract

Nutrition education, framed within Supplemental Nutrition Assistance Program Education (SNAP-Ed) guidance, was provided to SNAP-eligible shoppers at community supported agriculture (CSA) sites in Michigan where SNAP nutrition incentives were accepted. An evaluation was conducted on data sources from sites where the CSA Food Navigator program was implemented to assess the delivery of nutrition education, understand the needs and experiences of SNAP-eligible shoppers, and measure behavioral outcomes. A multi-phase, mixed-methods design incorporated (1) outcome surveys with SNAP-eligible shoppers at participating CSA sites; (2) open-ended feedback surveys from CSA site staff; (3) nutrition educator activity logs; (4) a semi-structured nutrition educator focus group; and (5) semi-structured focus groups with SNAP-eligible shoppers. In phase one, descriptive analysis was completed on the quantitative data and constant comparative analysis was completed on the qualitative data. In phase two, these data were collated into case reports for respective CSA sites; then, a cross-case analysis was performed. In phase three, statistical tests were performed on SNAP-eligible shoppers’ survey data to assess outcomes against a nationally representative sample of nutrition incentive program participants. Results indicate significantly higher fruit and vegetable consumption among shoppers relative to SNAP incentive participants nationally. Key qualitative themes were (1) relating over transacting: investing in multi-level relationships, (2) personalizing engagement and experiential nutrition education, (3) activating social–ecological spheres to promote changes in access, and (4) enhancing education support and resources for accessibility. The findings have practical implications to enhance the delivery and impact of CSA-based nutrition education.

## 1. Introduction

Lack of affordable food often manifests in concerning rates of household food insecurity (10.2%, 2021; 12.8%, 2022; 13.5%, 2023) in the United States (U.S.) [1]. People with very low or low food security face disparate health outcomes; for example, the rate of clinical hypertension for adults in food-insecure households was 21% higher than peers of the same age, education, and income [2]. The Supplemental Nutrition Assistance Program (SNAP) is a federal program that, through a monetary benefit, offsets the harms of food insecurity and promotes nutrition security by making food, including nutritious foods, more accessible [3]. Participating in SNAP has been shown to reduce food insecurity; a nationally representative survey of SNAP households found that 65% of those new to SNAP were food insecure, and that percentage decreased to 53.9% of households after participating in SNAP for six months [4]. SNAP participation has also been shown to conditionally reduce effects of hypertension and improve self-reported health [2,5]. While one of the benefits of SNAP participation is a positive change in food security status, there is less evidence indicating that SNAP participation positively influences nutrition security status [6,7,8].

To help people eligible for SNAP eat healthily on a limited budget and be physically active, the federally funded Supplemental Nutrition Assistance Program Education (SNAP-Ed) program provides evidence-based nutrition education, multilevel interventions, and community and public health approaches to improve nutrition and prevent obesity [9]. SNAP-Ed approaches follow the social–ecological model (SEM) which accounts for the ways multi-level factors (e.g., individual, familial, community, organizational, policy) may influence health promoting behaviors [10,11]. SNAP-Ed has demonstrated success across the nation among youth and adults in improving healthy eating, supporting participants in stretching their dollars for health promoting foods, increasing health promoting physical activity behaviors, and reducing sedentary behaviors [12,13,14]. For example, a national sample of SNAP-Ed participants reported a 40% improvement in fruit intake, a 34% improvement in vegetable intake, a 23% improvement in physical activity, and 41% improvement in shopping behaviors [14]. Researchers have demonstrated that increases in fruit and vegetable consumption are linked to reduced odds of diabetes and hypertension, respectively [15].

To further intervene in food and nutrition insecurities for SNAP beneficiaries, a federal program, the Food and Nutrition Incentive Program [(FINI), eventually renamed the Gus Schumacher Nutrition Incentive Program (GusNIP)], was initiated in 2014 to provide incentives for the purchase of more fruits and vegetables [16]. Over 2019–2023, grantees reported shifts in the food access landscape, improved household food security, increased daily fruit and vegetable intake, and increased fruit and vegetable expenditures [17,18,19,20,21].

### 1.1. Literature Review

The evidence-based benefits of providing incentives or subsidies for purchasing fresh fruits and vegetables through CSAs has grown. One randomized controlled trial provided participants in the intervention group with $300 to subsidize the cost of a CSA share and provided those in a control group with $300 cash, with the only restriction being that they could not purchase a CSA share that season. At baseline, 39% of participants reported receiving SNAP, and the CSA offered additional discounts for SNAP recipients separate from the $300 subsidy [22]. Researchers found that participants in the subsidized intervention CSA group demonstrated significant improvement in total Healthy Eating Index scores [22]. Food insecurity improved for both groups, but with a greater improvement for the subsidized intervention group as compared to the control [22]. Another project that included CSA shoppers eligible for SNAP or Special Supplemental Nutrition Assistance Program for Women, Infants, and Children (WIC) and CSA subsidies demonstrated a significant increase in intake for 11 of 30 different vegetables [23]. While the total mean intake of the provided vegetables increased significantly from before to immediately following the CSA season, intake returned to pre-CSA levels three months after the end of the season [23].

Nutrition programs in Michigan have the benefit of the state’s agricultural bounty. Michigan is the nation’s leading grower of asparagus, tart cherries, dry edible beans like black and navy beans, and squash [24]. The state is also among the top producers for many other specialty crops, from apples and blueberries to carrots and celery [24]. Double Up Food Bucks (DUFB), a matched dollar-for-dollar benefit program used in Michigan for the purchase of fruits and vegetables using SNAP, demonstrated that greater length of participation in the program was associated with reports of improved food security and greater fruit and vegetable intake [25]. Those who participated for seven months or longer reported eating more cups of fruits and vegetables daily [25]. This aligns with an evaluation of nutrition incentive program participants from across the U.S., which also demonstrated that sustained participation (more than 6 months) was associated with significant improvements in fruit and vegetable intake as well as food security [26].

While nutrition incentives are beneficial, there remains a need for evidence-based integration of nutrition education at CSA sites where SNAP-eligible shoppers redeem their benefits, in order to support CSA participation and other positive behavioral outcomes [27,28]. For example, one randomized controlled trial of the Farm Fresh Foods for Healthy Kids program implemented across four states reported that the effects of nutrition education combined with a cost-offset CSA for children and caregivers of low-income households had mixed results [28,29,30]. Evaluators of the program found a statistically significant increase in fruit and vegetable intake for caregivers but not children [28]. While the intake effects did not last past one CSA season, caregivers reported sustained improvement in cooking attitudes and self-efficacy, as well as in the inclusion of vegetables in children’s snacks and for dinners [29]. A key reported benefit of the nutrition education classes was that of receiving recipes for unfamiliar produce and new ways to prepare familiar produce; additional benefits were learning new cooking and preservation techniques, among others [30]. Reported barriers highlighted schedule conflicts, although participants appreciated when classes were scheduled to coincide with CSA pickup, and childcare for young children would have been helpful [30].

A different pilot of nutrition education at a CSA based in New York provided nutrition education through recipe cards, preparation and storage tips, and health information [31]. A nutrition booth was set up so participants could ask questions to a registered dietitian [31]. Participant surveys demonstrated the recipe cards were well received, there was significant improvement in knowledge about how to prepare vegetables, and the number of types of vegetables consumed increased significantly from pre- to post-, and there was an increase in the estimated volume of vegetables eaten in the last month, but that increase was not statistically significant [31].

### 1.2. Michigan Food Security and Intervention Context

#### 1.2.1. Michigan Food Security Context

Food security and health concerns are also evident in Michigan. The most recent data on Michigan’s food insecurity status show that rates have worsened over time: 15.4% in 2023 (~63% of Michiganders qualified for SNAP benefits) compared to 14.2% in 2022 (~56% of Michiganders qualified for SNAP benefits) and 11.7% in 2021 (~58% of Michiganders qualified for SNAP benefits) [32]. A 2023 survey of the general population of Michigan adults found that 19% reported fair or poor general health and 37% reported they had high blood pressure, with the rates for both measures worsening at lower household incomes [33,34,35]. Adult fruit and vegetable consumption, measured in Michigan in 2021, also worsened at lower household incomes; 68.9% of adults with household income less than $20,000 reported eating at least one vegetable a day while that increased to 86.7% for adults with household income at $75,000 or more [33].

According to the most recent data, Michigan’s population is less diverse than that of the U.S. as a whole, as measured by the 2020 Census Diversity Index (Michigan 45.2%, U.S. 61.1%), although it is home to many population groups [34]. In 2024, 14.1% of Michiganders were Black or African American, 6% were Hispanic or Latino, and measured for the first time in the Census in 2020, Michigan had the second largest population of people who reported Middle Eastern and North African (MENA) descent in the U.S. [36,37]. Serving the varied needs of people across Michigan requires attention to the 2020–2025 Dietary Guidelines for Americans, with particular attention to the guideline to “Customize and Enjoy Food and Beverage Choices to Reflect Personal Preferences, Cultural Traditions, and Budgetary Considerations” [38].

#### 1.2.2. Intervention Context: Michigan Farm to Family: CSA Food Navigator Program

Michigan Fitness Foundation (MFF), an organization that aims to improve fruit and vegetable intake and food security, and strengthen collaborations, among other goals, is currently a GusNIP grantee and contributes to increased food security through Michigan Farm to Family: CSA (MF2FCSA). MF2FCSA is a program that provides technical assistance to staff at community supported agriculture (CSA) sites (i.e., farms, farmers markets, and service organizations) that want to accept SNAP. Then, shoppers with SNAP can use their benefits to purchase boxes of fresh, Michigan grown produce at MF2FCSAs at a predetermined frequency, typically weekly during the summer growing season. The program includes a nutrition incentive for SNAP shoppers; they pay approximately 25% of the cost using SNAP, and grant funds reimburse the remainder of the CSA cost to the vendor. The year-over-year growth of MF2FCA sites [2021 (*n* = 8), 2022 (*n* = 15), and 2023 (*n* = 20)] and year-over-year growth in patronage by shoppers [2021 (*n* = 446 *) 2022 (*n* = 781 *), and 2023 (*n* = 825 *)] demonstrates persistent value for this particular nutrition incentive program (*n’s are an estimate based on raw data).

MFF is also a state SNAP-Ed implementing agency and awards grants to Michigan-based regional and local community partners to implement SNAP-Ed programming, where SNAP-eligible individuals live, work, learn, play and shop in their respective communities. This includes programs that address barriers to health (e.g., limited access to vegetables). In 2024, MFF partners delivered SNAP-Ed programming in 51 counties and demonstrated a 41% increase in vegetable consumption and a 22% increase in food security status among adult participants [13].

In the first year of the MF2FCSA program (2019), shoppers had not yet been exposed to nutrition education but expressed a need for it. To meet the needs of SNAP-eligible shoppers in CSA sites, MFF used a co-designed approach to pair the MF2FCSA program with SNAP-Ed to deliver nutrition education; as a result, the first iteration of the CSA Food Navigator program was developed.

The CSA Food Navigator program was piloted in 2021 by matching three MFF SNAP-Ed grantee organizations (“partners”) with local CSA sites participating in MF2FCSA. Trained SNAP-Ed nutrition educators (“Food Navigators”) developed and carried out needs-based nutrition education plans. A key learning from this formative year was that flexible nutrition education (e.g., food demonstrations and tastings, recipes) was a better fit for SNAP-eligible shoppers and within the CSA setting than more time intensive education (e.g., classes). Based on these learnings, in 2022, a CSA Food Navigator Playbook was developed to provide guidance, tools, and resources to help partners implement nutrition education in the CSA setting. The Playbook was modeled after the Farmers Market Food Navigator (FMFN) program playbook, which was designed by MFF for nutrition educators to serve as Food Navigators at farmers markets [39].

Currently, the CSA Food Navigator Playbook provides Food Navigators with resources and tools to deliver the program, from planning through implementation. Examples of Playbook resources include prompts to use when starting early conversations with CSA staff, along with tips and tools to help the Food Navigator become familiar with the CSA model. Additionally, the Playbook guides the Food Navigator to assess SNAP-eligible shoppers’ needs to plan relevant nutrition education activities. The Playbook also provides guidance and information for offering in-person nutrition education with fidelity; featured recipes and useful information about produce commonly distributed in CSAs in Michigan; tips for program promotion through social media, social marketing, and other communication channels; and suggestions for supportive community change work. While the iterations of the CSA Food Navigator program have not formally named the SEM as the underlying theory driving behavior, many factors of the SEM are present in the program and alluded to in the iterations of the Playbook.

The Playbook was developed to provide a framework for activities and a variety of materials to select from when providing nutrition education. This was important to ensure that the program could be effectively used by Food Navigators at CSAs throughout Michigan. In 2023, SNAP-Ed nutrition education was delivered via the CSA Food Navigator program in MF2FCSA sites for at least 5 weeks and up to 12 weeks. To support partners and Food Navigators, MFF has provided orientation and training for the intervention. In 2023, training sessions were provided through two synchronous virtual sessions on Zoom in May prior to program implementation. Trainings were recorded and made available for later viewing. The first session provided an overview of the CSA Food Navigator program, including an overview of the MF2FCSA program, a description of the MF2FCSA and SNAP-Ed collaboration, and an outline and timeline of key activities. The first session was structured as a question-and-answer session for partners considering CSA Food Navigator program implementation. The second session provided a thorough orientation to the CSA Food Navigator program details and Playbook content.

### 1.3. Aims of This Evaluation

While there are examples of nutrition education programs delivered at CSA sites, the models of delivery have distinct design and delivery approaches, and the outcome behaviors measured and sustainability of behavior change described in the literature have thus far been mixed. This evaluation aims to fill a need for additional models to be presented in the literature. It examines the outcomes of a co-designed nutrition education intervention delivered with SNAP-eligible MF2FCSA shoppers through SNAP-Ed using the CSA Food Navigator program. By adding to the body of literature, this evaluation can contribute to learning about what program elements and approaches contribute to success.

The purpose of this evaluation was to answer three questions: (1) How do fruit and vegetable consumption, food security, and self-rated health differ between CSA Food Navigator program participants and a national sample of SNAP incentive shoppers? (2) What measurable changes were observed in the nutrition security status of SNAP-eligible shoppers exposed to the CSA Food Navigator program at MF2FCSA sites? (3) To what extent did CSA Food Navigator program implementation at MF2FCSA sites benefit SNAP-eligible shoppers and CSA staff?

## 2. Materials and Methods

### 2.1. Methods

We followed a mixed-methods and multi-phase evaluation approach. All data collection activities took place between May and September 2023 and were approved under [(Farmers Market Food Navigator Program: Key Stakeholder Perceptions and Program Outcomes 210325 date of approval: 23 March 2021), (institution name and project number removed for peer-review purposes and will be added after notification of acceptance and before publication)].

#### 2.1.1. SNAP-Eligible Shopper Outcome Survey

SNAP-eligible shoppers who purchased produce from MF2FCSA sites where SNAP-Ed was implemented (*n* = 8 sites with *N* = 375 total SNAP-eligible shoppers) completed an outcome evaluation survey (*n* = 67 pre-, *n* = 99 post-surveys). This survey included questions about food and beverage consumption (a ten-question assessment developed from the National Cancer Institute [NCI] Dietary Screener Questionnaire [DSQ]), food security (the 6-item United States Department of Agriculture [USDA] Food Security Status module), and self-reported health status (one item, Would you say that in general your health is poor, fair, good, very good, or excellent?), which represent the Participant-Level Core Metrics designed by the Center for Nutrition and Health Impact [40]. Ordinal frequency responses to the DSQ were recoded and converted to cup equivalents of fruits, vegetables, and fruits and vegetables combined based on age- and gender-specific coefficients and portion sizes provided by NCI [41]. From the USDA module, food security status was recoded into “High food security” (0), “Marginal food security” (1), “Low food security” (2–4), and “Very low food security” (5–6) and dichotomized into “food secure” (0–1) and “food insecure” (2–6) based on individual item responses.

A question set of brief screeners developed by Calloway et al. [42,43] measured nutrition security status, healthfulness choice, dietary choice, and absorptive capacity. Using the suggested screening scores/coding outlined by Calloway et al. [42,43], respondents were scored as having “low” or “sufficient” measures of nutrition security and food insecurity resilience.

Additionally, the length of program participation, program experience, and sociodemographic characteristics (i.e., race, ethnicity, gender, age) were included in the survey to further contextualize factors that influence consumption behavior.

Shoppers were recruited to complete the survey by staff working at each CSA site. Shoppers were recruited at two time points: the start of the summer season (“pre”, May-June) and the end of the summer season (“post”, July-August). Surveys were administered through an online Qualtrics link with the option for paper surveys when requested [44]. Upon completion, respondents were offered the option of receiving a $10 electronic gift card; collection of information (i.e., email address) to administer the gift card was embedded in an additional Qualtrics survey to avoid linking personally identifiable information with outcome survey responses. Demographic variables of pre and post survey participants along with site of participation were used to iteratively identify matches; a total of *n* = 41 matched pairs were confirmed.

Survey links were coded with a CSA designation indicator to facilitate matching SNAP-eligible shoppers to respective CSA sites. If paper surveys were used, an evaluation staff member entered the responses into the respective Qualtrics link.

#### 2.1.2. SNAP-Eligible Shopper Focus Groups

SNAP-eligible shoppers were recruited to participate in a focus group by self-selecting at the end of the shopper outcome survey. Interviews were used in lieu of a focus group participation based on SNAP-eligible shopper availability. A total of 29 SNAP-eligible shoppers from MF2FCSA sites participated in either a focus group or interview.

The focus group guide was semi-structured and designed to be responsive to goals, objectives, and implementation practices. Shopper focus group questions included those on the enrollment process in the CSA program, inclusion in CSA site activities, program strengths and areas for improvement, fruit and vegetable consumption, needs for and engagement with nutrition education, and CSA communications. If an interview was necessitated the focus group guide was followed. Examples of some of the questions asked were, “What suggestions or feedback do you have for us to enhance the overall experience for current and future CSA customers?”; “What has the CSA program done to provide you with nutrition education, if at all?”; and “What changes to your dietary habits or food choices have you made because of the nutrition education received through the CSA program?”

Focus groups (or interviews, as necessitated by shopper availability) were administered through Zoom [45]. Participants were asked to give written informed consent prior to the engagement. Consent forms were disseminated through email and collected electronically via electronic signature. Technical assistance was provided over the phone for those who needed support to complete the form. Additionally, technical assistance was provided online via Zoom by sharing the link to the consent form using the Zoom chat feature. After all written consents were obtained, the lead evaluator asked participants to give verbal consent before proceeding with evaluation activities.

#### 2.1.3. Nutrition Educator Activity Logs

Food Navigators documented implementation of their activities, observations, and implementation experiences on weekly activity logs. Activity logs were uploaded to a secure cloud-based file management system for storage.

#### 2.1.4. Nutrition Educator Focus Group

Food Navigators were engaged in a post-season focus group. This focus group included questions about the nature of the CSA site, facilitators of shopper engagement, successes and challenges, techniques for relationship building, and identified needs for related community change work. The lead evaluator obtained informed verbal consent from participants and the second evaluator documented assertions before evaluation activities were conducted.

The focus group guide was semi-structured and designed to be responsive to goals, objectives, and implementation practices. The focus group was facilitated through Zoom [45].

#### 2.1.5. CSA Site Staff Process Survey

CSA site staff were hired by and/or were farmers of one of the eight CSA sites, and were engaged in a post-season open-ended survey. This survey included questions about the customer orientation process, communication with customers, customer engagement and community-building, and supporting the experience and needs of SNAP shoppers. Examples of some of the questions asked were, “How do you involve CSA customers in the produce selection decision-making process?”; and “What strategies do you use to communicate and build relationships with customers, outside of CSA pick-up days?”

Surveys were administered through an online Qualtrics link [44]. Survey links were coded with a CSA designation indicator to facilitate the matching of CSA staff to respective CSA sites.

#### 2.1.6. National GusNIP Sample

A nationally representative sample was used for data analysis [21]. These data (*N* = 9157) represent all Participant-Level Core Metrics surveys completed by participants in GusNIP-funded nutrition incentive programs and summarized by the GusNIP Nutrition Incentive Program Training, Technical Assistance, Evaluation, and Information Center (NTAE). Surveys were gathered from GusNIP-funded programs (*N* = 56) in the U.S. from 1 September 2022, to 31 August 2023. The national GusNIP sample included data from our local program; however the local sample only comprised approximately 1–2% of the national dataset. Although this introduces a minor violation of the assumption of independence, the small proportion contributed is unlikely to meaningfully bias test statistics. Therefore, national estimates were treated as fixed benchmarks for all statistical comparisons.

### 2.2. Analysis

Analysis was conducted in three iterative phases. Phase one case reports were generated for individual CSA sites where SNAP-Ed was delivered using descriptively analyzed quantitative data and qualitative themes. Phase two involved a cross-case analysis of these case reports for further themes across CSA sites where SNAP-Ed was delivered. Phase three used statistical tests to assess changes in our sample before and after the intervention and to compare our sample with a national sample. For context, the following headings in the report reflect the content in the results section: customer demographics (race, ethnicity, gender, age, length of participation in a CSA), self-rated health, fruit and vegetable consumption, food and nutrition security, customer experience, qualitative findings, and considerations for future implementation.

In phase one, individual case reports were developed through quantitative and qualitative data analysis that took place in R and Dedoose, respectively [46,47]. Survey data were cleaned and descriptives produced for each CSA site and time point (i.e., pre, post, and pre–post change among matched surveys). Focus group and/or interview recordings were professionally transcribed verbatim. Activity logs and open-ended survey responses were treated as transcripts. Two evaluation staff followed a grounded theory informed by the Charmaz constructivist approach [48,49], and one more senior member of staff, trained in qualitative analysis, coded the transcripts. All transcripts were coded inductively starting with line-by-line coding to generate emergent categories, and then with focused coding applied to categories [48,49]. After each stage of analysis, critical consensus-building discussions were held to explore definitions of codes and content of memos to build upon and/or refine categories. After analysis, results and findings were summarized into case reports, one for each respective CSA site. The case reports were shared by email with local partners to ensure that the summaries were representative of the local context as a member checking activity. Feedback from local partners and MFF staff were applied to the case reports to generate a final version of the case study report. Both evaluators believed that the separate components of the MF2FCSA model contributed to health promoting behaviors and positive changes in the food system. Therefore, discussion-based and email-based member checking was used to test those believed assumptions of evaluators. Once member checking was completed and feedback was applied, they were finalized and disseminated.

In phase two, a cross-case analysis was conducted on the individual case reports to build the evidence for the CSA Food Navigator program. This led to the development of one comprehensive case study. Case reports were uploaded to and analyzed in Dedoose to facilitate line-by-line and focused coding between the two evaluators. Each case report was treated as a transcript and analyzed following the same analytic approach in phase one: a grounded theory analysis informed by the Charmaz constructivist approach (i.e., line-by-line, focused coding, member checking) [48,49]. However, additional MFF staff were included in member checking since they had many years of familiarity with MF2FCSA or the CSA Food Navigator program (i.e., administrators, planners, resource development). First, the staff were emailed a copy of the report to review. Then, a face-to-face conversation during a CSA Food Navigator staff meeting took place to finish the member checking activity. Feedback was especially key in contextualizing food and nutrition security summaries and interrogating the framing of best practices produced during the analysis. The survey data were compiled into one dataset and analyzed. Descriptive analysis was conducted on SNAP-eligible shoppers’ responses to develop results. Mean differences in outcomes were generated from pre to post. Cross-case analysis activities took place between October 2023 and January 2024.

In phase three, the final phase of analysis, statistical tests were selected based on the type and distribution of the data to appropriately assess changes in our sample before and after the intervention and compare our sample with the National GusNIP sample. Since multiple statistical tests were conducted on the same samples, Bonferroni adjustments were applied to the *p*-values to minimize the overall probability of making a Type 1 error while ensuring findings were more robust. All analyses were conducted using R [47].

To examine pre- and post-intervention changes in our sample, paired *t*-tests were conducted on daily fruit and vegetable consumption [50]. This test is appropriate for assessing mean differences in matched observations [50]. The Wilcoxon signed-rank test were selected to assess changes in food security status and self-rated health, which were measured on ordinal scales [51]. This test is designed to detect whether the distribution of responses to an ordinal variable differs between paired groups [51]. The McNemar’s test was used to compare the proportions of individuals experiencing low nutrition security, healthfulness choice, dietary choice, and absorptive capacity before and after the intervention [52]. The McNemar’s test is appropriate for testing differences in proportions in paired measurements of nominal variables [52].

To compare mean daily fruit and vegetable consumption in our sample with national averages, a one-sample *t*-test was conducted [50]. This test is appropriate when evaluating whether the mean of a sample differs significantly from a known or hypothesized population mean [50]. In this case, the means from the national sample served as the reference values. To compare the proportions of food secure and food insecure individuals in our sample and the national sample, the Chi-square test was employed [53]. This test is appropriate for examining differences in proportions across independent groups for variables with nominal response options [53]. To assess differences between our sample and the national sample in self-rated health, a Mann–Whitney U Test was conducted [54]. This test is designed to detect whether the distribution of responses to an ordinal variable differs between independent groups [54].

## 3. Results

### 3.1. Survey Results of SNAP-Eligible Shoppers Exposed to Nutrition Education via the CSA Food Navigator Program in CSA Food Navigator Sites

To assess the outcomes of shoppers exposed to nutrition education via the CSA Food Navigator program in MF2FCSA Food Navigator sites, a series of quantitative analyses were conducted on the shopper behavior survey data. Key results for fruit and vegetable consumption, food security and nutrition security status, and self-reported health status are summarized below. Comparisons were made between matched pairs from pre to post in our sample. The time between pre–post responses ranged from 4.5 weeks to 8.6 weeks. As an additional comparison, we also examined differences between our sample and the National GusNIP sample.

#### 3.1.1. Pre–Post Comparisons

In Table 1, the mean cup equivalents of daily fruit and vegetable consumption, daily fruit only consumption, and daily vegetable only consumption measured before and after the intervention were compared using paired *t*-tests. The tests revealed no statistically significant changes in fruit and vegetable consumption (fruit and vegetable, t(34) = 0.90, *p* = 0.999; fruit, t(34) = 0.207, *p* = 0.999; vegetable, t(34) = 0.66, *p* = 0.999).

A Wilcoxon signed-rank test (Table 2) was conducted to compare the distribution of food security status among Michigan SNAP-eligible shoppers before and after the intervention, while accounting for the ordinal nature of food security status (Very low food security, Low food security, Marginal food security, High food security). The results did not indicate a statistically significant change in food security status (V = 48.5, *p* = 0.999).

A Wilcoxon signed-rank test (Table 3) was conducted to assess whether the distribution of self-rated health changed after the intervention, while accounting for the ordinal nature of self-rated health (Poor, Fair, Good, Very Good, Excellent). The results did not indicate a statistically significant change (V = 95, *p* = 0.999).

McNemar’s tests (Table 4) were also conducted to compare the proportion of individuals in our sample experiencing low nutrition security, healthfulness choice, dietary choice, and absorptive capacity before and after the intervention. The results did not indicate statistically significant changes in these variables (nutrition security, Χ^2^(1) = 1.78, *p* = 0.999; healthfulness choice, Χ^2^(1) = 0.00, *p* = 0.999; dietary choice, Χ^2^(1) = 0.00, *p* = 0.999; absorptive capacity, Χ^2^(1) = 0.01, *p* = 0.999).

#### 3.1.2. Comparisons with National GusNIP Sample

**Figure 1:**
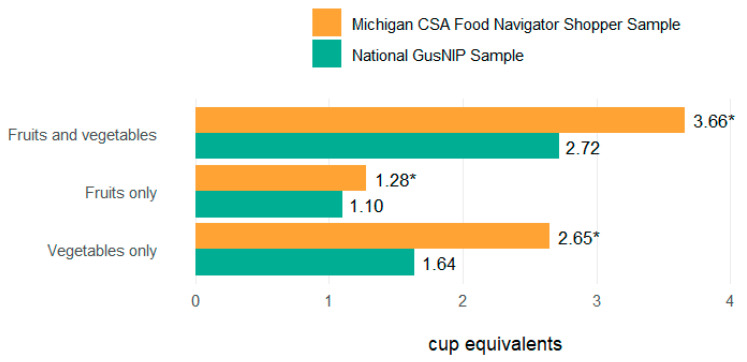
Comparisons with National Sample Mean Fruit and Vegetable Consumption. Note: Ordinal frequency responses to the 10-item Dietary Screener Questionnaire (DSQ) were converted to cup equivalents of fruits and vegetables. Michigan CSA Food Navigator Shopper Sample (*n* = 80–81). National GusNIP sample (*n* = 7689–7851). * Indicates that mean cup equivalents consumed by Michigan SNAP-eligible shoppers was significantly different from the national mean, based on a one-sample *t*-test.

In Figure 1, we compared the mean cup equivalents of daily fruit and vegetable consumption, daily fruit only consumption and daily vegetable only consumption in our sample to the national averages in 2023. The tests revealed statistically significant differences for each comparison (fruit and vegetable, t(79) = 5.56, *p* < 0.001; fruit, t(80) = 2.81, *p* = 0.031; vegetable, t(79) = 8.78, *p* < 0.001). The consumption of fruits and vegetables (+0.94 cup equivalents), fruits only (+0.18), and vegetables only (+1.01) was higher in our sample compared to the national sample.

**Figure 2:**
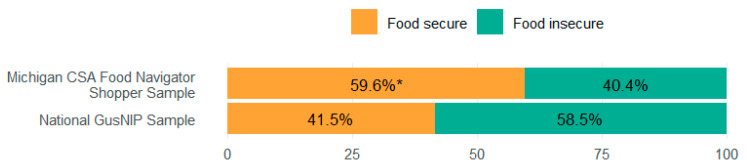
Comparison with National Sample Food Security Status. Note: Responses to the 6-item USDA Food Security Status module were dichotomized into “food secure” (0–1) and “food insecure” (2–6). Michigan CSA Food Navigator Shopper Sample (*n* = 99). National GusNIP sample (*n* = 5838). * Indicates that proportion of food secure individuals in the Michigan SNAP-eligible shoppers’ sample was significantly different from the national sample, based on Chi-square test.

A Chi-square test (Figure 2) was conducted to compare the proportion of food secure and food insecure individuals in our sample with the national sample. There was a statistically significant difference (Χ^2^(1) = 12.314, *p* = 0.002); specifically, the proportion of food secure individuals was higher in our sample compared to the national sample (+18.1%).

**Figure 3:**
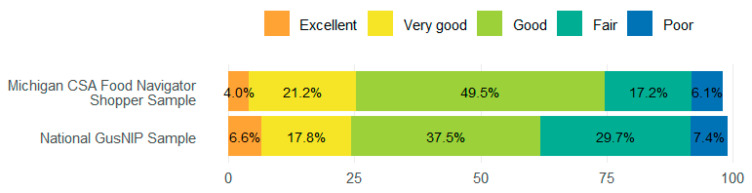
Comparison with National Sample Self-Rated General Health. Note: Survey question: Would you say that in general your health is poor, fair, good, very good, or excellent? Michigan CSA Food Navigator Shopper Sample (*n* = 99). National GusNIP sample (*n* = 5445). No significant changes were found.

A Mann–Whitney U test (Figure 3) was conducted to assess whether the distribution of self-rated health differed in our sample and the national sample. The results did not indicate a statistically significant difference (U = 241,042, *p* = 0.294).

### 3.2. Qualitative Findings

Qualitative case reports were analyzed to describe the collaborators’ (i.e., CSA staff, Food Navigators, SNAP-eligible shoppers at MF2FCSA sites) experiences with the multi-level MF2FCSA and CSA Food Navigator program. Four primary themes were developed: (1) relating over transacting: investing in multi-level relationships, (2) personalizing engagement and experiential nutrition education, (3) acting within the SEM to promote changes in food access, and (4) enhancing education support and resources for accessibility. The themes are described below and contextualized in Table 5. The multi-level collaboration addressed variables relating to economics, education, and social and community context. Analysis of the narratives found that the CSA Food Navigator program promoted food and nutrition security, as well as social connectedness.

#### 3.2.1. Theme 1: Relating over Transacting: Investing in Multi-Level Relationships

Multi-level collaborators invested in one another by taking the time to develop trusted relationships. Collaborators worked together and that contributed to the shift in the construct of currency from ‘money for goods’ to ‘building trust for transformative behavior change’; namely, the currency between CSA site staff and shoppers shifted from transactional to relational. Thus, relationship became the currency that facilitated SNAP-eligible shoppers to meet their food access needs in a more accessible and dignified way. CSA site staff have demonstrated that food access should be humanized and relational since they made the decision to belong to MF2FCSA and CSA Food Navigator programs. Relationship building informed the perception of the high quality of customer service and that was key to the success of SNAP-Ed delivery of the CSA Food Navigator program. The economic situation, nutritional behavior, and well-being of shoppers were impacted by the relational approach taken by CSA site staff and Food Navigators, which simultaneously influenced the shopper’s experience,

*“… staff… never made me feel like (pauses) unworthy, I guess is maybe a way to say it”.*

[CSA Shopper, Focus Group #9, Participant #6, Case #1]

*“It brings joy to me [having access at the CSA site] because I can’t afford produce all the time. I mean, I’d love to, but I don’t. It’s hard to go and buy a bag of carrots or celery or whatever and not look at the price and say ‘oh, I can’t do that today.’ Without this program I don’t know if I would even eat a vegetable. And that’s being honest”.*

[CSA Shopper Interview #4, Case #2]

The relational approaches used by the multi-level collaborators were apparent in most cases among Food Navigators, shoppers, and CSA site staff. Shoppers reported that CSA site staff and food navigators contributed to a welcoming environment and worked together to provide a comprehensive experience. For example, in some cases, Food Navigators introduced shoppers to CSA site staff and CSA site staff introduced shoppers to farmers, which facilitated a sense of community connection. Food Navigators reported that investing in a relational, reflective approach to delivering nutrition education inspired shoppers’ behavior change. One shopper explained how in-depth communication helped support them in linking CSA box items to recipes. These relational communication strategies employed by Food Navigators gave shoppers the sense that they were cared for by Food Navigators. The level of care influenced shoppers to return to the CSA site, ask nutrition related questions, and/or feel connected to the community through these collective efforts:

*“Yeah, so there’s an email sent out each week a couple of days… we’re able to know a little bit about the staff member that sent it, giving us details about them, what they like, what they do around the farm. And then also as far as the food, it lists… the certain farm that it came from, and there are also recipes on the email as according to what food is in the box”.*

[CSA Shopper, Focus Group #1, Participant #3, Case #3]

In one case, the Food Navigator who delivered education in two different types of CSA sites (semi-urban cases: one market, one healthcare facility) talked about how navigators were open and genuine, which helped shoppers reciprocate openness about food celebrations and challenges. This Food Navigator and navigators in several other cases reported that shoppers affirmed the relational investments made when shoppers came back and shared their food stories. Similar approaches were used in other sites and the trust between navigators and shoppers blossomed. A summary of these experiences is depicted through the lens of Food Navigators and shoppers below, or can be found in Table 5,

*“[the shopper] stopped by and said they liked the smoothie. They also said they’d made the kale pesto from 7/6/2023 and liked it very much, using it on crackers and in sandwiches.”*

[Document Review, Food Navigator Log, Case #1]

*“One of my favorite things was when they returned to the next pick-up with stories of how they would make the recipes I provided them, at home with success!”*.

[Document Review, Food Navigator Log, Case #4]

*“…that personal connection and you know building those relationships… you have the same people coming back over and over. They see that they can trust you… if you can make that eye contact, be that friendly first face and, you know, ask them… if they would try this recipe… that interaction really motivates them, motivates them to try cooking. They come back and they tell you what they’ve tried [cooking] you know the spark and the curiosity and making that personal connection, really, I think maybe made the… biggest difference in keeping them [shoppers] curious and making them brave about cooking and trying food”.*

[Food Navigator, Focus Group Participant #3, Case #3]

Shoppers in several cases described relational, conversational experiences with CSA site staff. A shopper in one case, where poor weather inhibited travel, reported that the CSA site staff went above and beyond to deliver the CSA box to their home. The shopper reported that this gesture displayed a depth of care. This depth of care was also evidenced by shoppers who stated CSA site staff knew them by name. This developed a sense of familiarity and shoppers felt noticed. An urban CSA was noticed for friendly CSA staff, which made an impression on the shopper and promoted an attitude of gratitude. Shoppers explained that the relational, conversational approach was expressed through follow-up communications with shoppers after a visit. Another CSA site staff reported their commitment to putting relationships first and curating a family-oriented CSA environment. The relational, conversational approach contextualized norms for engagement which led to shoppers feeling cared for and repeating those tactics with their peer shoppers. The following quotes further explicate how multi-level relationships became a currency of their own:

*“When we had a storm, and we couldn’t get our boxes, they brought my box… You don’t find a lot of people doing that stuff. They went above and beyond”.*

[CSA Shopper Interview #4, Case #2]

*“They [site staff] knew everyone that came in. They [site staff] already had a relationship with them…” and, “Everyone [staff and shoppers] knew everyone there and they [shoppers] would share recipes and what to do with collard greens and trying different things. And then they looked at some of the recipes that I had”.*

[Food Navigator Focus Group Participant #8, Case #5]

*“The first [orientation] phase includes paperwork sent through email and the second phase includes an email, text message, and phone call. During the second phase customers are prompted to ask questions to improve clarity in the customer experience. Over the season, the farmers, who are the site staff, get to know each customer by first name—through those efforts—a familial customer interaction transpires.”*

[CSA Site Staff, Survey Respondent, Case #6]

#### 3.2.2. Theme 2: Personalizing Engagement and Experiential Nutrition Education

Educating and engaging shoppers requires preparation, communication, and monitoring to enhance the shopper experience. Since the CSA environment is dynamic and shoppers’ needs are personal, Food Navigators had to invest more time and effort than anticipated in preparation and planning to be most effective. When Food Navigators arrived at the CSA sites, active listening was applied simultaneously with nutrition education delivery. Navigators noticed that the coupling of prior planning with responsive, contextualized nutrition education that felt personal helped shoppers practically apply the education in the home environment (e.g., lesson content, method of delivery, mix of resources, type of materials).

Shoppers, in most cases, shared that the intentional effort put forth by Food Navigators to engage and personalize the educational experience made an impact on them, especially the hands-on activities. The personalized engagement and delivery of nutrition education made it easier for shoppers in most cases to express gaps in knowledge, such as learning about the produce in multiple ways (e.g., in-person, on paper, tastings, plain language, pictures, words) or learning different methods of preparation or cooking familiar but abundant items or unfamiliar items in their box. As a result, shoppers reported increased nutritional knowledge, shifts in attitudes (from hesitant to brave) towards unfamiliar produce, and enhanced willingness to try new things (i.e., produce, recipes, and cooking techniques). Several shoppers and Food Navigators explicated the benefits of engagement and experiential nutrition education in the quotes below and in Table 5:

*“We had a few people [shoppers] that were really hard of hearing, so they [shoppers] were really appreciative of the all the printed resources”.*

[Food Navigator Focus Group Participant #2, Case #2]

*“… one lady had come back and said, you know, I’m really thankful that you put those things [plain language, highly visual materials] in the box because I would have never asked… You know, they were really simple, easy instructions that I was able to follow along and you know, now I’m not afraid to cut into a butternut squash or… an acorn squash”.*

[Food Navigator Focus Group Participant #2, Case #2]

*“There was one time during the summer where we had an option to receive a plant to grow our own vegetables… I was so nervous… it was a pepper plant and I had never grown peppers and they [the Food Navigator] really took the time to explain it, how to water it, how to care for it… they never made me… feel like I was asking the wrong question, or it was a silly question”.*

[CSA Shopper, Focus Group #1, Participant #3, Case #4]

One Food Navigator, whose primary audience consisted of people 60 years or older, described how they were consistently able to plan for education delivery beforehand. which allowed them to emphasize engagement while implementing nutrition education and, in turn, promoted social connectedness between shoppers. For example, shoppers this Food Navigator interacted with shared generational stories about food. Guided by Food Navigators, these shoppers used the nutrition education gathering to conduct informal peer-to-peer nutrition education through their generational stories. Additionally, this population, which had more potential years of exposure to foods, still wanted to learn. The following quotes give deeper context to this case and also explain the essence of the ways Food Navigators, broadly, prioritized engagement in nutrition education:

*“One thing I did I think because I had an audience… I would always arrive early and set up… whatever food was going to be in the CSA… They [shoppers] loved that. They talked about the recipes…”*

[Food Navigator Focus Group Participant, #1, Case #6]

*“I really think this [nutrition education] is niche for the older generation, to get people [the older generation of shoppers] together. They [shoppers] would often share different things that were in their boxes… what they we’re going to make… it was just very sweet to see”.*

[Food Navigator Focus Group Participant #1, Case #6]

*“The chickpeas did not come from the farm, but the tomatoes and the cucumbers did. And to be having a food tasting with someone in their 70 s that says, what are these? And we say chickpeas. And we talk about protein and to introduce somebody to a really valuable protein at age 70 felt like a big win. Simple, affordable, healthy”.*

[Food Navigator Focus Group Participant #1, Case #6]

#### 3.2.3. Theme 3: Acting Within the Social–Ecological Model to Promote Changes in Access

One important finding was that everyone (i.e., MFF staff, CSA site staff, local SNAP-Ed partners, and Food Navigators), in their own way, described how MF2FCSA and CSA Food Navigator were aligned with the tenets and constructs of the SEM. Respectively, everyone described specific factors and actions that optimized the ways they benefitted from the program design and/or delivery in the context of the SEM. Everyone worked together to make food more accessible and affordable and, by design, increased shopper autonomy over food access through affordability, acceptability, choice, and knowledge.

The findings of this theme are presented by the various SEM spheres, starting with factors associated with the intrapersonal, interpersonal, and social spheres, then finishing with factors associated with the organizational sphere. Contextually, no narratives directly described federal, state, or local policy, factors so the representation of the data stops with the organizational sphere; however, policy level factors (SNAP guidance included electronic benefits transfer acceptance at CSA sites) were already intact. Conceptually, shoppers in most cases reported that intrapersonal change (thoughts and perceptions about vegetables changed, especially unfamiliar vegetables) was facilitated by interpersonal factors (Food Navigators delivered nutrition education), community level factors (CSA sites accepted nutrition incentives), and societal and organizational level factors (CSA staff collaborated with MFF and local partners, normalizing nutrition education at CSA sites).

In some cases, participants (i.e., shoppers, CSA site staff, and Food Navigators) contextualized the ways intra- and inter-personal facilitators as well as community approaches. represented conceptual alignment with the SEM, such as increased opportunities for accessible produce and factors that influence consumption of fresh produce:

**Intra-and Inter-personal Level Factors**

*“I would say… like trying new vegetables and stuff like that, things that I normally wouldn’t buy have got me out of my comfort zone a little bit and I am really happy about that”.*

[CSA Shopper, Interview #3, Case #4]

*“… the thing I would highlight the most… the variety of produce… I think it actually helped to improve the health in my family, compared to what we were eating before the CSA box”.*

[CSA Shopper, Focus Group #5, Participant #1, Case #4]

*“I never would have bought like kohlrabi and Swiss chard and a few other [unfamiliar] things. And the same thing. You can just chop them up and throw them in anything that you already were making anyway. And on the rare week when we have too much—we almost never have too much, but if we do, then I freeze stuff, so we have it in the wintertime”.*

[CSA Shopper, Focus Group #3 Participant #3, Case #1]

*“It’s the variety and the quality… I have noticed and it has enhanced my diet and my life because it introduces you to other flavors and you say to yourself, “Oh, I definitely want to keep eating that” … and it made me want to actually eat them more regularly… I have [chronic] disease, and I also have lost [weight], so I am always on the lookout to how do I eat more, better in general for health and longevity kind of thing”.*

[CSA Shopper Focus Group #3, Participant #2, Case #1]

**Community and Organizational Factors**

*“CSA members are invited to participate in any of the classes and services offered, shop at the farm stand, and/or volunteer with the organization in whatever capacity they are interested in”.*

[CSA Site Staff, Survey Respondent, Case #8]

*“the [CSA site name] offered a very similar program in a very different environment where there was a farm setting in the greenhouses on the hospital campus that people from a very broad, broad, cross section of the community would come in to select produce, they could opt to select or not select the produce that had already been picked from whichever farmers brought produce in that week and could cut fresh flowers”.*

[Food Navigator Focus Group Participant #3, Case #3]

In a few cases, participants contextualized social and organizational approaches that upheld alignment to the SEM and normalized consumption of produce. To facilitate normalization of produce consumption at the social and organizational levels, several sites took action to meet needs. One site developed a year-round farm stand, another implemented a “round-up” system to accept donations that incentivized affordable produce, and yet another site introduced a blemish basket, where they made edible produce that might have had a visual abnormality (e.g., a bruise) available for free. One site leveraged their position with the farm to help champion the meeting of shopper’s needs and preferences, which offset staffing shortfalls in organizational bandwidth. Social and organizational approaches are illustrated in the following quotes:

*“The farm we are currently partnering with does not provide client customization to the CSA shares they deliver. However, they allow our organization to influence/choose the general items that our shares get out of what they have available for the week. We base those choices on general client preferences reflected through other purchases or if we receive any consistent verbal feedback”.*

[CSA Site Staff, Survey Respondent, Case #7]

*“Since I am not having to setup and man a booth at a market or run a storefront, the costs are much lower than retail”.*

[CSA Site Staff, Survey Respondent, Case #6]

*“After someone verbally commits to participating, we reach out to them and schedule a day/time for them to come in for a 1–1 orientation and paperwork… go over the details of what they are committing to and a schedule for the summer… They then receive a reminder the week that the CSA is starting and weekly reminders thereafter”.*

[CSA Site Staff, Survey Respondent, Case #7]

*“… a lot of times you go to a food bank and they—a lot of the stuff is not stuff that you should be eating. I mean it’s not conducive to a [medically tailored] diet. So having access to all of these vegetables and then being able to… buy the CSA box… really makes it so much more possible and affordable for me to have—to get a really good diet with lots of fresh veggies. And it’s that’s a wonderful thing”.*

[CSA Shopper, Focus Group #4, Participant #2, Case #7]

#### 3.2.4. Theme 4: Calling for Enhancements: Support for Delivering Education and Resources for Accessibility

Food Navigators, in most cases, reported that training sessions and technical assistance provided by MFF that focused on relationship building were supportive, and Food Navigators associated these training sessions with influential delivery of the CSA Food Navigator program. Food Navigators, in some cases, strongly implied that more training and resources would be beneficial in supporting preparation and planning. Contextually, Food Navigators found it challenging to deliver effective nutrition education if they did not have enough materials, resources, and/or protected time to plan nutrition education prior to getting to the CSA site. This challenge was explicated by the dynamics of having multiple responsibilities and perceiving each to be high priority/high impact. With the lack of protected time to prepare and/or obtain materials, resources, and lesson plans, Food Navigators perceived, in some cases, that the level of support they received from MFF and/or CSA site staff was weak.

Food Navigators who had a stronger level of support from CSA site staff reported that they found that they had a greater all-around impact on shoppers. In most cases, Food Navigators attributed stronger support to communication factors (e.g., timeliness, consistency, relevance, breadth/depth) being provided, or not, by CSA site staff. Food Navigators who experienced weaker levels of support wanted CSA site staff to be more communicative about the contents of the produce boxes, while CSA site staff that provided stronger levels of support demonstrated the impact it had on their delivery of education. In one case, the Food Navigator in a rural CSA site had consistent and timely communication with the CSA site staff, who described the produce contents of the box. Stronger levels of support created opportunities for the Food Navigator to be responsive to customer feedback through adjusting activities for learning levels, type of, and delivery of, nutrition education (e.g., cooking instructions), and modifying ingredients on recipe cards, as described below:

*“… my CSA provider would contact throughout the week, and [they] would let me know what would be in the box for the week and I always would tailor my recipes and what I was bringing that week to what was in the box, which was always a hit”.*

[Food Navigator, Focus Group Participant, #4, Case #4]

*“… [the CSA site staff/farmer] always gave a good estimate [of what would be in the box] and then we would try and… come up with… the unique recipes or… what Michigan Harvest of the Month had… just really correlating those [estimates from farmer and recipes on Michigan Harvest of the Month] …and keeping in touch with our CSA provider like on a weekly basis”*

[Food Navigator, Focus Group Participant #2, Case #2]

Navigators expressed the need for resources to meet the needs of specific populations. The CSA Food Navigator program was designed to deliver nutrition education to adult shoppers and materials primarily in English; however, Food Navigators encountered needs for additional resources to meet specific population needs. The importance of having materials available in various languages and for various age groups was highlighted in some cases. Food Navigators noticed that their reach, in some cases, included youth. In other cases, navigators reported that some shoppers preferred languages different than that which the Food Navigator typically used. While Food Navigators expressed the need for materials, such as recipes in various languages, they also expressed that their skillset did not position them to create such materials. The quotes below contextualize the support and accessibility factors outlined:

*“And when the kids came with their parents, that was also really cool because they were a little bit hesitant to try most of the snacks. But when they did, they almost always loved them”.*

[Food Navigator, Focus Group Participant #5, Case #8]

*“I think a large need that came up at our site specifically was a language barrier because we had a lot of Spanish speaking individuals coming up”.*

[Food Navigator, Focus Group Participant #6, Case #7]

*“I would like some more recipe ideas you know and a little more flexibility… Not to only use the recipes found in the Michigan farm to family or in the playbook but often these people would share recipes with one another from their childhood, or things that their mother made.”*

[Food Navigator, Focus Group Participant #1, Case #6]

## 4. Discussion

This evaluation adds to the small body of literature that highlights the value of offering nutrition education at CSA sites where shoppers use their SNAP benefit, and sometimes subsidies, to purchase produce. Overall, this multi-level, collaborative, and relational approach to improving food access, uptake of vegetable consumption, and self-reported health via the CSA Food Navigator model points to the benefit of aligning interventions that include nutrition education and fruit and vegetable subsidies with the SEM [11]. Addressing food access at multiple levels (i.e., subsidy and nutrition education) was viewed favorably by shoppers, Food Navigators, and CSA site staff. Collaboration was key to sound and responsive nutrition education delivery. The relational approach includes the shopper, the Food Navigator, and the CSA site staff and contributed importantly to the findings. The findings suggest that nutrition education enhances the shopper experience at these food access points. Nutrition education was not measured as a distinct variable in this evaluation or, generally, in the cited literature in the discussion and conclusion, so the results should be interpreted with care.

The nuance of the differences in the quantitative results can be considered alongside the qualitative data. The qualitative findings, especially the shopper and Food Navigator narratives, provide insight into the quantitative findings. Quotes that explicate a favorable relationship between exposure to nutrition education via the CSA Food Navigator program and positive influence on health-related behavior and status changes are found in qualitative themes one and two, as well as in theme three, where the quotes are contextualized within the SEM. In themes one (relating over transacting: investing in multi-level relationships) and two (personalizing engagement and experiential nutrition education), participants reported improved vegetable consumption and food resource management. Shoppers also described the ways in which they stretched food dollars and shared produce-based recipes with family or friends. A few shoppers also contextualized the relational aspect of the program as an influence on self-reported health status, including “feeling better” or describing weight loss. In theme three (acting within the social–ecological model promotes change in access), shoppers reported improvements in vegetable consumption and self-reported health status for themselves and their family. The qualitative findings suggest that, due to improved access to quality foods and support from Food Navigators via relational nutrition education, shoppers’ behavior and status were changed.

The evaluation sought to examine CSA shoppers’ self-reported health outcomes via survey results from the local sample members who were exposed to nutrition education. Evaluators were not able to confirm statistically significant changes in behavior and status (i.e., vegetable consumption, food and nutrition security, self-reported general health) among the local sample pre- to post- intervention. Time may have been a factor in this lack of significance. There was a range in time taken between when pre- and post- surveys were taken, and the number of times nutrition education was delivered across sites varied. The ideal exposure to nutrition education that predicts behavior change or perception of health in CSA sites is unknown. Another factor is that shoppers could opt in to a weekly or monthly CSA subscription and that option may have influenced exposure to nutrition education, the amount of produce in the box, and when the surveys were taken. Finally, despite previous research showing a change in food security when subsidized CSA boxes are provided, this sample did not demonstrate such a change [22]. After the intervention, there was a small decrease in the percentage of individuals in our sample experiencing “Very low food security” (−4.9%), an increase in “Low food security” (+12.2%), a decrease in “Marginal food security” (−7.3%), and no change in “High food security”. This suggests that the availability and diversity of produce in the box, as well as timing, could be improved.

Another aim of the evaluation was to compare self-reported health outcomes from the local sample of shoppers exposed to the CSA Food Navigator program with the national sample of shoppers who were part of the GusNIP program, who had all responded to a survey consisting of the same core metrics. The one-sample *t*-tests demonstrated a statistically significant difference between the local sample and national sample in fruit and vegetable consumption together, fruit consumption alone, and vegetable consumption alone. Additionally, Chi-square tests indicated a significant difference in food security status. These analyses confirmed that our local sample, who were exposed to SNAP-Ed nutrition education via the CSA Food Navigator program, had more favorable outcomes around fruit and vegetable consumption compared to the national sample. There was not a significant difference in self-reported general health status. Our data are a small subset within the national analysis, differing only in the addition of nutrition education, and supporting the use of national estimates as valid benchmarks with minimal concern for overlap bias.

The difference between our model and the national model is the inclusion of nutrition education, which may help explain the statistically significant differences between samples. The details of the qualitative data in themes one and two explain the ways in which adding nutrition education via the CSA Food Navigator model enhances the relational aspect of the program. For example, narratives explain how relationships led to personalized engagement and responsive education. The presence of a Food Navigator may also explain the statistical differences between the local and national sample, since shoppers reported the benefit of learning about unfamiliar produce and being able to translate that into consumption, i.e., tasting, even enjoying, unfamiliar produce. In Michigan, there are many specialty crops, and the abundance of these crops might have influenced the box contents. As previously explained, differences may be a result of frequency of box pick-up and/or subscription model (e.g., weekly vs. bi-weekly vs. monthly), which would impact exposure to vegetables and exposure to a Food Navigator.

This evaluation was designed to account for the experiences of shoppers, Food Navigators, and CSA staff. Overall, the experiences and benefits were positive across all participant groups. As noted, the qualitative context represented in themes one, two, and three from the local sample contrasts with the survey results reported from the local sample (i.e., vegetable consumption, food security status, and self-rated general health status). The qualitative data from themes one and two help to explain and contextualize the statistically significant differences that favored the local sample as compared to the national sample. The third and fourth themes point to theory and practices that could strengthen the continuation of the positive experiences and benefits described by the participants.

Theme one (relating over transacting: investing in multi-level relationships) highlights the value of multi-level and relational collaboration in developing needs-based solutions for increasing food access. Theme two (personalizing engagement and experiential nutrition education) expands upon and further contextualizes the relational approach, underscoring the delivery of experiential learning by Food Navigators for shoppers. Experiential learning approaches were key to building relationships and the uptake of nutrition education. The multi-level, collaborative relationships between Food Navigators and shoppers contributed to shopper satisfaction, shoppers’ behavior change, and sometimes change in familial behavior. Transformative relationships are dependent on trust and, since social connection, collaboration, and relationships are trust building activities, this could explain the nuance in themes one and two.

In theme three (acting within the social–ecological model promotes changes in access), evaluators found that participants (shoppers, Food Navigators, CSA site staff) described their active approach to maintaining multi-level and collaborative relationships. Theme three illustrates how and in what ways participants acted within and between the levels of the SEM to promote food access. For example, shoppers explained how Food Navigators presented them with education which had ripple effects on personal behavior changes as well as familial changes. CSA site staff explained that it was important to use their role to leverage food access; specifically, CSA site staff helped to expand the knowledge and social networks of shoppers by introducing them to farmers who had contributed to the CSA box. Additionally, staff leveraged their role to promote shopper choice by taking on the responsibility of gathering shoppers’ preferred produce items and explaining those desires to the farmers who supplied produce. The findings from theme three give justification to the formal framing of the design of the CSA Food Navigator program in the SEM, since this evaluation found that participants naturally follow the tenets and constructs of the model to improve and/or enhance food access. This theme was likely present since the collaborators involved had a common interest and shared vision that was predicated on policy change, invited a systems approach, and changes in the environment were bound to occur, all making food access easier for shoppers.

In theme four (calling for enhancements: supports for delivering education and resources for accessibility), Food Navigators articulated a need for additional technical assistance and resources aimed at supporting them in implementing the CSA Food Navigator program for all participants in a needs-based manner. The narratives summarized factors that contributed to high levels or low levels of support and illustrated how low levels of support from MFF and/or CSA site staff could potentially inhibit the ways in which Food Navigators could contribute to effective nutrition education delivery. Given that Food Navigator narratives across themes highlight a desire to deliver education in a relational, responsive, and needs-based way, this theme further substantiates the importance of a co-designed Playbook.

The results and findings of this evaluation were both similar to and different from existing research in various ways. The studies by Chan et al. (2025), and Seguin-Fowler et al. (2021) [28,31] did not compare their sample to a national sample; however, Chan et al. (2025) [28] did employ a pre–post collection and Seguin-Fowler et al. (2021) [31] collected surveys at three time points (baseline, measured in Spring, time one, measured in Fall, and time three, follow-up a year later). Chan et al. (2025) [28], as in our evaluation, did not find significant increases in the volume of vegetables consumed, while Seguin-Fowler et al. (2021) [31] did find a favorable difference, but the changes [31] were not sustained when measured at the third timepoint. Of note, the weeks of program delivery were similar across these studies (12 weeks and 9 weeks, respectively) [28,31] and ours (range of weeks from 5 to 12). In the Seguin-Fowler et al. (2021) [31] study, where nutrition education was delivered for 9 weeks, significant changes in vegetable consumption volume were found; this suggests that it is plausible to find changes among shoppers who participate in the CSA Food Navigator program in the future, given a more defined number of weeks and/or the provision of cooking equipment. The results from this evaluation also differ from those of Seguin-Fowler et al. (2021), who found increased food security among adult CSA participants engaging in nutrition education [31]. Changes among this sample [31] should be studied further to discern how the intervention differed from the CSA Food Navigator program; one intervention difference that might explain the differences in outcomes would be the inclusion of guardian–child dyads in the educational process. The results from our evaluation also differ from those of Seguin-Fowler et al. (2021), who found increased food security among adult CSA participants who engaged in nutrition education [31].

One additional study, that of Izumi et al. (2020), that included nutrition education for adults in a subsidized CSA model demonstrated results different from ours; they reported significant change in self-rated general health status [55]. In this study, participants were recruited from a clinical setting and characterized as having no or limited access to health insurance; perhaps the participants in this case perceived greater gain in their self-reported health. While we did not find statistical significance for this variable, the focus group participants did specifically report the benefits of participation on their health. We cannot assert that our sample reported changes in health due to the nutrition education only, and posit that it is likely due to the multi-level, collaborative, and relational approach to education delivery, and making food easier to access. Similarly, Izumi et al. (2020) [55] drew similar conclusions about the potential for CSAs that accept subsidies and offer nutrition education to promote changes in health status.

The CSA Food Navigator program model, and similar program models, should continue to accentuate the power of relationships [56] within the context of the SEM. Relationships and experiential learning were key to delivery and uptake of nutrition education in our study; similarly, Martin et al. (2022), who used Photo Voice to engage participants, suggested that multi-level and collaborative factors were associated with participants’ increased ability to try new foods and improved health outcomes [57]. Our study, paired with others [56,57], highlights the value of multi-level and relational collaboration in developing needs-based solutions for increasing food access [11,56,57,58,59,60]. For example, Verfueth et al. (2023) asserted that a focus on relationships between CSA members (*n* = 16 interview participants) and farmers could promote personal well-being, familial well-being and food literacy [56]. They [56] further assert that food well-being can be achieved via relationships that foster a sense of belonging, which, in their case, was achieved by becoming a CSA member. An active approach to maintaining multi-level and collaborative relationships within and between social–ecological spheres of influence contributes to food access via delivery of nutrition education [11,58,59,60]. Sharkey and Smith (2023) [58], who used mixed methods, reported improved intake, though non-significant, of vegetables among parent–child dyads exposed to nutrition education in a cooking setting. While they did not find significant differences via survey, qualitative methods, similar to this evaluation, described the intervention as influential regarding intra- and inter- personal change. In our study, multi-level, collaborative relationships between Food Navigators and shoppers contributed to shopper satisfaction, shoppers’ behavior change, and sometimes change in familial behavior [56,58,59,60].

An emphasis should be placed on supporting the time and effort Food Navigators require to translate training and technical assistance into practice. Food Navigators, more often than shoppers in this evaluation, expressed barriers to implementing nutrition education. One barrier faced by Food Navigators, cultural relevance, was also described in other studies [59,60] and was framed as a barrier due to preferred language. The qualitative themes from our evaluation align with [59,60] in other ways. For example, our evaluation found relationships between farmers and participants to be important and so did Lu et al. [59] and Garrity et al. (2023) [60]. Furthermore, our evaluation and that of Garrity et al. (2023) [60] reported additional facilitators for engaging in a CSA, including collaboration, nutrition education for self, championing nutrition education by sharing learning with family and/or friends, and social interactions with farmers. To this end, the Playbook should include more resources for Food Navigators to enhance shoppers’ experience in a culturally relevant manner. Additionally, resources should be developed for CSA site staff to promote a deeper understanding of the multi-level needs of collaborators in the CSA Food Navigator model.

To ensure meaningful delivery of nutrition education, transformative change in shoppers, and sustainability of the program’s impact on shoppers and the food system, future evaluations should focus on additional implementation regarding science variables in the CSA environment. It is key to understand how or in what ways an individual or mix of concepts, such as dosage, environment, shoppers’ characteristics, and/or theory, might explain statistically significant changes in consumption, food and nutrition security status, and self-reported health.

### Limitations

The local sample was small, which may inhibit generalizability. While we did make a comparison of pre and post data from our local sample, we suspect that the pre and post time points were not far enough apart for shoppers to show change in some of the measures, especially in the case of nutrition security. One limitation faced was access to publicly available raw data from the comparison data set. As such, our analytical options were limited to using national means and counts to compare with our local behavior data. We aimed to buffer this by using statistical analysis approaches that could improve rigor of the test on given measures. We did not compare local and national nutrition security data, as these data were not collected in the core measures at the national level.

Additionally, the CSA Food Navigator program has been designed to allow flexibility for program implementation in response to local context and CSA operations. For example, some CSA sites provided shopper choice, while others provided boxes with predetermined contents and some models allow shoppers to subscribe weekly or monthly. Program guidance did not require a specific frequency of nutrition education activity delivery, which could be a limiting factor in assessing the effectiveness of nutrition education. This may limit the sensitivity of the measuring of food security and vegetable consumption.

## 5. Conclusions

The CSA Food Navigator program demonstrated the benefits of a multi-level collaboration for CSA shoppers. The benefits were found in the qualitative themes, i.e., improved food access using a CSA model that is designed to make it easier for shoppers to pick up produce in their community, pay for produce at a reduced cost, consume more produce, and gain knowledge about produce that might have been unfamiliar, in order to promote consumption. Food Navigators, who delivered nutrition education in CSA sites where SNAP was accepted and provided nutrition incentives, effectively developed trusting relationships and responded to the needs of CSA shoppers in promoting food access. To enhance nutrition education delivery at CSA sites, entities could benefit from clearly articulating communication expectations for the multi-level players and balance fidelity with responsiveness to benefit shopper’s experience, promote shopper’s behavior change, and ensure improved food security status.

The multi-level collaboration and relational approach strengthened the case for the CSA Food Navigator program in the SEM; having a theory-based approach complements a program already favored by its intended audience—shoppers. While the pre–post results from the local sample did not meet statistical significance, the post-only comparison between the local and national sample gives cause for more exploration using quantitative methods to ascertain what might drive such a measurable change. Future practices of the CSA Food Navigator program should focus on aligning implementation strategies (e.g., frequency of education, frequency of pick-up, and timing of education and pre–post surveys) while remaining flexible to the local community context. Researchers may consider exploring whether differences persist in measurable outcomes (e.g., vegetable consumption) due to shopper choice at CSA sites; for example, in some sites, shoppers had more autonomy over what fruits or vegetables made up their share, while in other sites some shoppers may have had a box prepared for them. Shoppers choice or lack thereof may determine the quantity of vegetables in a box and therefore be a predictor of volume of consumption. 

## Figures and Tables

**Table 1:** Pre–Post Comparisons Mean Fruit and Vegetable Consumption.

	Pre (sd)	Post (sd)
Fruits and vegetables	3.76 (1.68)	3.58 (1.37)
Fruits only	1.29 (0.52)	1.28 (0.63)
Vegetables only	2.72 (1.22)	2.65 (0.86)

Note: Ordinal frequency responses to the 10-item Dietary Screener Questionnaire (DSQ) were converted to cup equivalents of fruits and vegetables. Michigan SNAP-eligible shoppers, matched pairs (*n* = 37). No significant changes were found.

**Table 2:** Pre-Post Comparison Food Security Status.

		Pre (*n*)	Post (*n*)
Food security status	Very low food security	22.0% (9)	17.1% (7)
	Low food security	26.8% (11)	39.0% (16)
	Marginal food security	17.1% (7)	9.8% (4)
	High food security	34.1% (14)	34.1% (14)

Note: Responses to the 6-item USDA Food Security Status module were recoded into “High food security” (0), “Marginal food security” (1), “Low food security” (2–4), and “Very low food security” (5–6). Michigan SNAP-eligible shoppers, matched pairs (*n* = 41). No significant changes were found.

**Table 3:** Pre-Post Comparison of Self-Rated General Health.

		**Pre (*n*)**	**Post (*n*)**
Self-Rated General Health	Poor	7.3% (3)	7.3% (3)
	Fair	29.3% (12)	12.2% (5)
	Good	29.3% (12)	51.2% (21)
	Very Good	22.0% (9)	22.0% (9)
	Excellent	12.2% (5)	7.3% (3)

Note: Survey question: Would you say that in general your health is poor, fair, good, very good, or excellent? Michigan SNAP-eligible shoppers, matched pairs (*n* = 41). No significant changes were found.

**Table 4:** Pre-Post Comparisons of Nutrition Security and Related Measures.

		Pre (*n*)	Post (*n*)
Nutrition security	Low	46.3% (19)	34.1% (14)
	Sufficient	53.7% (22)	65.9% (27)
Healthfulness choice	Low	34.1% (14)	36.6% (15)
	Sufficient	65.9% (27)	63.4% (26)
Dietary choice	Low	39.0% (16)	39.0% (16)
	Sufficient	61.0% (25)	61.0% (25)
Absorptive capacity	Low	43.9% (18)	47.5% (19)
	Sufficient	56.1% (23)	52.5% (21)

Note: Responses to brief 1- to 2-item screeners that measured nutrition security status, healthfulness choice, dietary choice, and absorptive capacity, developed by Calloway et al. [42,43], were scored as “low” or “sufficient”. Michigan SNAP-eligible shoppers, matched pairs (*n* = 41). * No significant changes were found.

**Table 5:** Qualitative Themes and Illustrative Participant Quotes.

Central Theme		Example Quote(s) Illustrating Themes	
Theme #1	Relating over Transacting: Investing in Multi-level Relationships	*“… to let [shoppers] know that that’s a normal thing [not knowing exactly what to do with the produce] … So that’s… where I came in and to make sure that they knew they weren’t alone”* [Food Navigator Focus Group Participant, #4, Case #4]. *“I am just always raving about the CSA box. I… definitely like… build my week around it and just kind of I’m like always talking about it to people I see because I think it’s something that really boosts my quality of life”* [CSA Shopper, Focus Group #9, Participant #4, Case #1]. *“I am enjoying the fresh vegetables and the fact that they’re so friendly. And it’s just I’ve got a lot of stuff frozen for this winter because of it”* [CSA Shopper, Focus Group #6, Participant #2, Case #6]. *“…my food dollars go further …the vegetables are fabulous… initially they had a meeting with just everyone talking about the CSA overall… and that was helpful because I hadn’t done this before… and answer all the questions that you had, and included in that meeting without being—singling people out who were going to use a Bridge card, the SNAP benefits, answering those questions… that helped”* [CSA Shopper, Focus Group #2, Participant #6, Case #3]. *“I love, love, love when [shoppers] come back the next week and I see them and they say ohh, I tried this at home, and it turned out great and my family really liked that. So, for me it’s a success. If they have taken something, I provided them with and used it successfully at home”* [Food Navigator Focus Group Participant #4, Case #4]. *“We were there throughout the summer and it was a fun experience… they [shoppers] loved their boxes that they got, and they always really loved when the recipe that we gave went along with what they had in their box and they could try something new that was a healthy option”* [Food Navigator Focus Group Participant #5, Case #8].	
Theme #2	Personalizing Engagement and Experiential Nutrition Education	*“And then you know the pleasant surprise when you’re having them [shoppers] try the tasting right there on the spot, and they’re surprised at particular ingredients in there (the tasting) or that you can actually make something delicious with collards without cooking it, just that interaction that, you know, that shows that sense that immediate moment of discovery and surprise. [That] was a wonderful thing that happens pretty often*” [Food Navigator Focus Group Participant #7, Case #1]. *“I have found myself doing more raw vegetables, vegetables that I have traditionally cooked and because of the recipes that are provided when we do the pick-up… so I’ve started eating more raw vegetables from some of those recipes that have been provided. One in particular, collard greens, I always cooked them. I mean … I didn’t know, you know, raw collard greens. And they provided a recipe and a sample of collard greens with a couple of the other vegetables from that box that week and that was special… and it was tasty”* [CSA Shopper, Focus Group #2, Participant #6, Case #3]. *“Well, I wasn’t sure on what some of the vegetables were because I had not done this. But [Food Navigators] explained everything, and they even gave us a little hand—what to do with it and how to cook it or freeze it. So, they’ve been very informative”* [CSA Shopper, Focus Group #6, Participant #2, Case #6].	
Theme #3	Acting within the Social–Ecological Model Promotes Changes in Access	Intrapersonal Level Factor	*“… so that [being part of the CSA] was new and a great experience. I got to have food… that I had never had experience with before… broadened my food—especially in produce, and veggies, and all of that. It just opened up my palette to new flavors and experiences…”* [CSA Shopper, Focus Group #3 Participant #2, Case #1].
		Interpersonal Level Factor	*“So, I am trying to just like snack on veggies now… I guess over the last few months… around the same time [as starting the CSA]… I lost [weight]… it’s just encouraging when you’re working out to be able to eat good… it makes me feel better, too, and just having [produce] available on hand all the time, too. Not that my daughter always wants to eat veggies, but she is a little more open to it, too”* [CSA Shopper Interview #3, Case #4].
		Organizational Level Factor	*“If there is a farmer here, we do an introduction [to shoppers], if their food is in that week’s box, and we engage with lots of customers in an effort to get them [shoppers] to know who is growing their food… We value the connection and want them to know each other”* [CSA Site Staff, Survey Respondent, Case #1].
		Community Level Factor	*“Well, … I’ve got lettuce and a nice tomato to put on it… I have also been working with a dietician to deal with the [chronic diseases and conditions] trying to lower my cholesterol… for me, it’s [the weekly CSA box] worked out really great because it just dovetails with what I am doing with the dietician”* [CSA Shopper, Focus Group #4, Participant #2, Case #7].
Theme #4	Calling for Enhancements: Support for Delivering Education and Resources for Accessibility	*“The fresh food is worth it. And it makes the kids want to eat more fruits and veggies… It’s helped to try different things, too. So, I got zucchini, I made zucchini bread, and had the kids try that for the first time. So that was fun”* [CSA Shopper, Focus Group #7, Participant #2, Case #4].	

## Data Availability

The datasets presented in this article are not readily available because the data are part of an ongoing study. Requests to access the datasets should be directed to bbode@michiganfitness.org.

## Abbreviations

The following abbreviations are used in this manuscript:

CSA	Community supported agriculture
FINI	Food Insecurity Nutrition Incentive
GusNIP	Gus Schumacher Nutrition Incentive Program
MFF	Michigan Fitness Foundation
MF2FCSA	Michigan Farm to Family Community Supported Agriculture Program
SEM	social-ecological model
SNAP	Supplemental Nutrition Assistance Program
SNAP-Ed	Supplemental Nutrition Assistance Program Education
U.S.	United States
USDA	United States Department of Agriculture
